# Physiological Significance of NAD Kinases in Cyanobacteria

**DOI:** 10.3389/fpls.2019.00847

**Published:** 2019-06-27

**Authors:** Yuuma Ishikawa, Maki Kawai-Yamada

**Affiliations:** Graduate School of Science and Engineering, Saitama University, Saitama, Japan

**Keywords:** cyanobacteria, *Synechocystis*, NAD, NAD kinase, metabolism

## Abstract

Unicellular cyanobacteria are thought to be the evolutionary ancestors of plant chloroplasts and are widely used both for chemical production and as model organisms in studies of photosynthesis. Although most research focused on increasing reducing power (that is, NADPH) as target of metabolic engineering, the physiological roles of NAD(P)(H) in cyanobacteria poorly understood. In cyanobacteria such as the model species *Synechocystis* sp. PCC 6803, most metabolic pathways share a single compartment. This complex metabolism raises the question of how cyanobacteria control the amounts of the redox pairs NADH/NAD^+^ and NADPH/NADP^+^ in the cyanobacterial metabolic pathways. For example, photosynthetic and respiratory electron transport chains share several redox components in the thylakoid lumen, including plastoquinone, cytochrome *b_6_f* (cyt *b_6_f*), and the redox carriers plastocyanin and cytochrome *c6*. In the case of photosynthesis, NADP^+^ acts as an important electron mediator on the acceptor-side of photosystem I (PSI) in the linear electron chain as well as in the plant chloroplast. Meanwhile, in respiration, most electrons derived from NADPH and NADH are transferred by NAD(P)H dehydrogenases. Therefore, it is expected that *Synechocystis* employs unique NAD(P)(H) -pool control mechanisms to regulate the mixed metabolic systems involved in photosynthesis and respiration. This review article summarizes the current state of knowledge of NAD(P)(H) metabolism in *Synechocystis*. In particular, we focus on the physiological function in *Synechocystis* of NAD kinase, the enzyme that phosphorylates NAD^+^ to NADP^+^.

## Introduction

NAD(P)(H) are important electron carriers, employed by all living cells in energy conversion. NAD^+^ and NADP^+^ (oxidized forms), or NADH and NADPH (reduced forms) act as oxidizing agents or reducing agents in electron-transfer steps in several metabolic pathways. It is thought that the presence of the non-phosphorylated forms (NAD^+^ and NADH) and phosphorylated forms (NADP^+^ and NADPH) make it possible for multiple redox reactions to coexist simultaneously within the cell (Alberts et al., 2002). In plants, intensive studies have been performed to clarify the importance of the individual NAD(P)(H) species in growth and stress responses (Dutilleul et al., 2005; Schippers et al., 2008; Takahashi et al., 2009; Sienkiewicz-Porzucek et al., 2010; Takahara et al., 2010; Bernhardt et al., 2012; Pétriacq et al., 2012, 2016; Maruta et al., 2016). The influence of quantitative changes in the NAD(P)(H) balance on plant growth has been elucidated by reverse genetics using knockout or overexpression strains. The *Arabidopsis old5* mutant harbors a mutation in the nuclear locus encoding the chloroplast-localized quinolinate synthase; the mutant exhibits increased NAD^+^ levels and early senescence phenotypes (Schippers et al., 2008). In contrast, overexpression in *Arabidopsis* of the cytoplasmic NAD synthase yields decreases NAD^+^ levels and accelerated senescence (Hashida et al., 2016). The *Arabidopsis nudx19* mutant harbors a loss-of-function in the nuclear locus encoding a NADPH pyrophosphohydrolase that localizes to the chloroplast and peroxisome; this mutant shows increased NADPH contents in whole cell extracts, but not increased NADP levels. In contrast, plants overexpressing the *Arabidopsis* chloroplast-localized NAD kinase (NADK2) show an increased NADP(H) pool (i.e., elevations in both NADP^+^ and NADPH concentrations). Interestingly, both plants (*Arabidopsis nudx19* mutant and NADK2-overexpressed transgenic plant) have been reported to exhibit enhanced carbon assimilation and tolerance to photooxidative stresses (Takahara et al., 2010; Maruta et al., 2016). The relationship between balancing of NAD(P)(H) and the physiological roles of these molecules in plants is complicated. This lack of clarity reflects the fact that plants have compartmentalized cell areas (chloroplast, mitochondria, vacuole, nucleus, etc.) within the cell. At present, experimental techniques do not permit measurement of the NAD(P)(H) contents of individual compartments in plant cells. As a result, there is no alternative to using quantitative results for the whole cell extracts.

**FIGURE 1:**
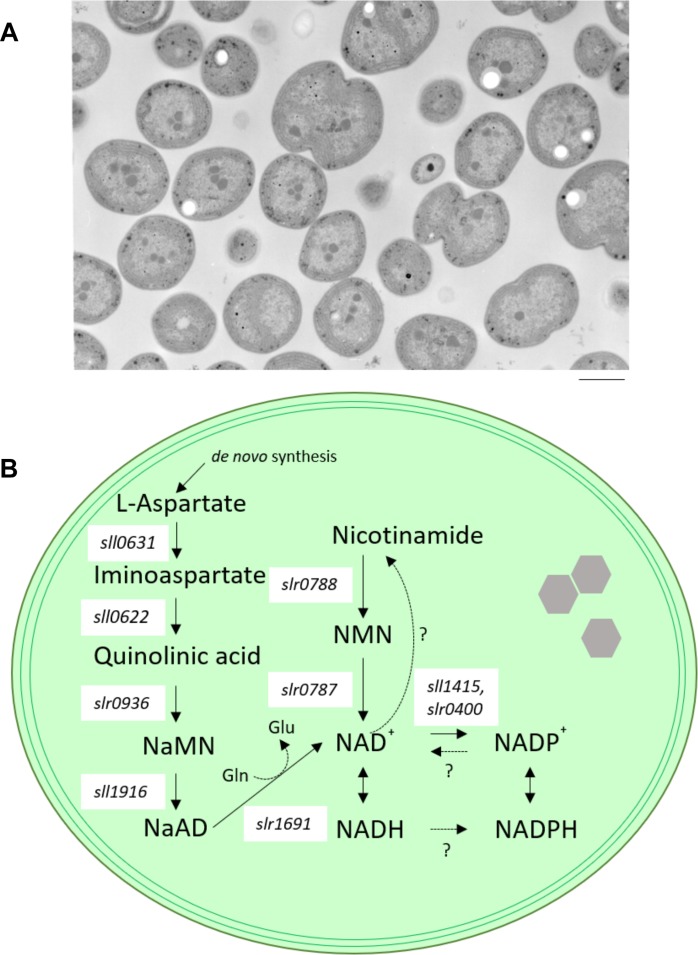
Model cyanobacterium *Synechocystis* sp. PCC 6803. **(A)** transmission electron microscope (TEM) images of wild-type cells of *Synechocystis* sp. PCC 6803. Bar indicates 1 μm. **(B)** Schematic diagram illustrating NAD^+^ biosynthesis in *Synechocystis* sp. PCC 6803. Enzymes contributing to NAD synthesis are the products of individual genes. Only NAD kinase (NADK), which catalyzes the phosphorylation of NAD^+^ to NADP^+^, is encoded by two separate genes (*sll1415* and *slr0400*) in *Synechocystis* sp. PCC 6803. NaMN, nicotinate mononucleotide; NaAD, nicotinate adenine dinucleotide; NMN, nicotinamide mononucleotide; Gln, glutamine; Glu, glutamic acid; *sll0631*, L-aspartate oxidase-encoding gene; *sll0622*, quinolinate synthetase-encoding gene; *slr0936*, quinolinate phosphoribosyl transferase-encoding gene; *sll1916*, nicotinate nucleotide adenyl transferase-encoding gene; *slr1691*, NAD synthase-encoding gene; *slr0787*, nicotinamide nucleotide adenyl transferase-encoding gene; *slr0788*, nicotinamide phosphoribosyl transferase-encoding gene.

On the other hand, cyanobacteria, which have comparatively simple cell structures, perform most metabolic reactions in a shared space (Figure 1A). Therefore, we expect that cyanobacteria are better suited to examining the direct effects of changes in NAD(P)(H) contents or balance. However, in cyanobacteria, there were (until recently) few reports on the use of the genetics of NAD(P)(H) metabolism for understanding the physiological roles of NAD(P)(H) species.

Cyanobacteria are the only prokaryotes capable of using light energy to perform plant-like oxygenic photosynthesis; these organisms often are used as production platforms in synthetic biology. For example, in recent years, research on biofuel and chemical production using cyanobacteria has been conducted. To enhance the productivity of these bacteria and the economic feasibility of bioproduction, most research in cyanobacteria has focused on increasing reducing power (that is, NADH or NADPH) by genetic improvement of cofactor metabolism (Hauf et al., 2013; Shabestary and Hudson, 2016; Wang et al., 2016; Zhou et al., 2016). The NADPH is involved in some cellular metabolism, e.g., the Calvin cycle, hydrogen biosynthesis, lipid biogenesis, amino acid biosynthesis and chlorophyll synthesis. Therefore, the molecular basis of NAD(P)(H) metabolism is crucial for planning a strategy to raise the yield of valuable substances.

In cyanobacteria, photosynthesis and respiration share some components of electron transport chain. So far, electron transport pathways in thylakoid membrane has been determined (summarized in Lea-Smith et al., 2016). In the case of photosynthesis, excitation energy trapping by PS II results in water splitting, and transport of electrons to the primary and secondary electron accepting quinone molecules Q_A_ and Q_B_, respectively (Barthel et al., 2013). Following double reduction and protonation, electron is released from Q_B_ into the plastoquinone (PQ) pool and delivers electrons to the cyt*b_6_f* complex (Kurisu et al., 2003). Cyt*b_6_f* transfers one electron to either plastocyanin or cytochrome, and these small soluble electron carriers donate electrons to P700^+^ (Duran et al., 2004). In addition to respiration, it is now reported that many respiratory components may participate in PQ pool reduction including NAD(P)H dehydrogenase-like complex type I (NDH-1), succinate dehydrogenase (SDH) and one to three NDH-2s (Mi et al., 1992; Howitt et al., 1999; Cooley et al., 2000; Ohkawa et al., 2000). Furthermore, the cytochrome bd quinol oxidase, encoded by cydAB, has been identified in thylakoid of *Synechocystis*. Cytochrome bd quinol reduces oxygen with electrons presumably taken directly from the PQ pool (Berry et al., 2002; Ermakova et al., 2016). Finally, the aa3-type cytochrome oxidase complex, encoded by coxABC, can accept electrons from plastcyanin/cytochrome c6 to produce the H^+^ gradient (Howitt and Vermass, 1998). Plants perform above mentioned electron transports (photosynthesis and respiration) in chloroplast and mitochondria. While, in cyanobacteria, these two redox pathways occur in a single cell compartment (Figure 1A), and share with a several components such as PQ, cyt*b_6_f* and plastcyanin/cytochrome *c6*. Currently, a central question has never addressed in cyanobacteria: how photosynthesis and respiration are regulated in a same membrane? In addition, most primary metabolic pathways, such as the Calvin-Benson cycle, the tricarboxylic acid (TCA) cycle, and glycolysis, also share the same cytosolic space (Knoop et al., 2010). Additionally, *Synechocystis* sp. PCC 6803 employ metabolic adaptative systems to provide alternative supplies of required substances. For example, under photoheterotrophic conditions (e.g., in medium containing glucose and 3-(3,4-dichlorophenyl)-1,1-demethylurea (DCMU), a compound that inhibits photosynthesis), and under low-light conditions (where illumination is too weak for photosynthesis to occur) in the presence of glucose, cyanobacteria utilize medium-supplied glucose as both an energy and carbon source, and generate NADPH through the oxidative pentose phosphate pathway to compensate for photosynthesis-derived NADPH (Narainsamy et al., 2013; Nakajima et al., 2014; Ueda et al., 2018). Based on these unique metabolic characteristics, cyanobacteria provide a unique tool for examining several relevant topics, including: (1) How do cyanobacteria control the amount of the redox pairs NADH/NAD^+^ and NADPH/NADP^+^ in mixed metabolism in the single compartment? (2) Which metabolic pathway is controlled by NAD(P)(H) under the various growth conditions in cyanobacteria?

This review focuses on the current state of knowledge of NAD(P)(H) metabolism in cyanobacteria. Notably, the physiological functions of NADKs, the enzymes that catalyze the synthesis of NADP^+^, are discussed.

## NAD^+^ Synthesis in Cyanobacteria

Several genes involved in NAD^+^ biogenesis have been identified by comparative genomics in a model cyanobacterium, *Synechocystis* sp. PCC 6803 (Figure 1B; Gerdes et al., 2006). Additionally, four of the NAD biosynthetic enzymes, *sll1916* (NaMNAT), *slr1691* (NADS/GAT), *slr0787* (NMPRT), *slr0788* (NMNAT) were biochemically characterized (Raffaelli et al., 1999; Gerdes et al., 2006). The state of knowledge of the process of cyanobacterial NAD^+^ biogenesis can be summarized as follows. Firstly, aspartate serves as a precursor of the *de novo* pathway. The conversion of aspartate to nicotinate mononucleotide (NaMN) is implemented via three consecutive reactions catalyzed by L-aspartate oxidase (nadB), product of the *Synechocystis* sp. PCC 6803 *sll0631* gene), quinolinate synthase (nadA), product of *Synechocystis* sp. PCC 6803 *sll0622*), and quinolinate phosphoribosyl transferase (nadC); product of *Synechocystis* sp. PCC 6803 *slr0936*). Next, NaMN is converted to nicotinate adenine dinucleotide (NaAD) by nicotinate-nucleotide adenylyl transferase (NaMNAT; product of the *Synechocystis* sp. PCC 6803 *sll1916* gene). Subsequently, NaAD is converted to NAD^+^ by amidation, followed by phosphorylation of NAD^+^ to NADP^+^. In this final step of NAD^+^ biogenesis in cyanobacterium, NAD synthase (NADS/GAT; product of the *Synechocystis* sp. PCC 6803 *slr1961* gene) requires glutamine as a source of an amide group for the ATP-mediated amidation reaction (Raffaelli et al., 1999). Such NAD^+^ synthesis pathway is consistent with all analyzed cyanobacteria (Gerdes et al., 2006) and *Arabidopsis thaliana*. *Arabidopsis thaliana* use a salvage pathway in which NaMN is synthesized from nicotinamide (Katoh et al., 2006; Wang and Pichersky, 2007). On the other hand, the routes for salvage or recycling of NAD^+^ vary significantly between cyanobacterial species (Gerdes et al., 2006). Moreover, *Synechocystis* sp. PCC 6803 possesses an alternative nicotinamide mononucleotide (NMN)-specific adenylyltransferase (encoded by *slr0787*), a novel bifunctional enzyme. Specifically, the N-terminal domain of the Slr0787 protein catalyzes NAD synthesis from NMN and ATP, while the C-terminal region of Slr0787 possesses Nudix hydrolase activity. Nudix hydrolases cleave an X-linked nucleoside diphosphate (where X is phosphate, sugar, nucleoside mono/diphosphate, etc.), an activity that is involved in the control of the cellular levels of metabolites and toxic compounds (Bessman et al., 1996). However, under standard growth conditions, the *slr0787*-deficient mutant shows no phenotype compared to wild-type cells (Gerdes et al., 2006). The presence of the bifunctional Slr0787 protein indicates that cyanobacteria have a special system for controlling NAD(P)(H) metabolism.

Furthermore, *Synechocystis* sp. PCC 6803 possesses a conversion nicotinamide phosphoribosyl transferase (NMPRT, the product of *slr0788*), which catalyzes nicotinamide to NMN. This observation indicates that *Synechocystis* sp. PCC 6803 can synthesize NAD^+^ from nicotinamide via *slr0787* or *slr0788*. In contrast, the *Synechocystis* sp. PCC 6803 pathways for recycling and consuming NAD^+^ and NADP^+^ remain to be clarified. Additionally, Gerdes et al. (2006) showed that active uptake of nicotinamide is absent in *Synechocystis* sp. PCC 6803. Therefore, we believe that it will be necessary to study the contribution of the NAD-consuming pathway (including NAD consumption by ADP-ribosylating enzymes (Silman et al., 1995), NADP^+^ phosphatase, and Nudix hydrolases) in order to fully understand the homeostasis of NAD(P)(H) in *Synechocystis* sp. PCC 6803.

## NAD Kinases in Cyanobacteria

NADP^+^, the phosphorylated form of nicotinamide adenine dinucleotide (NAD), plays an essential role in many cellular processes. Notably, NADP^+^ is an electron mediator in the linear electron transfer chain of photosynthesis, where the NADP^+^ serves as a final acceptor of electrons in photosynthetic organisms (Hurley et al., 2002). NADP^+^ also is involved in primary metabolic reactions, such as those of the oxidative pentose phosphate pathway for the synthesis of NADPH, and those of the TCA cycle. Notably, in primary metabolism, NADP^+^ is an essential co-factor for the enzyme activities of glucose-6-phosphate dehydrogenase (G6PDH), 6-phosphogluconate dehydrogenase (6PGDH), and isocitrate dehydrogenase (ICD) in *Synechocystis* sp. PCC 6803 (Muro-Pastor and Florencio, 1992). The *Synechocystis zwf* mutant, in which G6PDH is inactive, becomes bleached under photoheterotrophic growth conditions and under light-activated heterotrophic conditions (Jansen et al., 2010). Deficiency for the *icd* gene is known to be lethal in *Synechocystis* sp. PCC 6803 (Muro-Pastor et al., 1996). Thus, NADP^+^-related enzymes have fundamental, essential functions required for this cyanobacterium to maintain carbon metabolism.

*PntAB* (pyridine nucleotide transdehydrogenase) is yet another important factor of NAD(P): NAD(P)H redox homeostasis maintenance which is located in the thylakoid membrane in *Synechocystis* (Kamarainen et al., 2017). *PntAB* is an integral membrane protein complex coupling the oxidation of NADH and concurrent reduction of NADP^+^ to proton translocation across the membrane. *PntAB*-deficient mutant exhibits growth defects under low-light and day-night rhythm in the presence of glucose. On the other hands, there was no apparent difference between the *pntAB*-deficient mutant and WT under the mixotrophic condition (20 μE/m^2^/s). Interestingly, *sll1415*-deficient mutant, one of the *NADK*-deficient mutants in *Synechocystis*, and *pntAB*-deficient mutant showed the similar phenotype under the day-night rhythm in the presence of glucose and the mixotrophic condition (20 μE/m^2^/s) (Ishikawa et al., 2016, 2019; Kamarainen et al., 2017). Therefore, *pntAB* may maintain the NADPH pool with *sll1415* by unknown coordination system.

**FIGURE 2:**
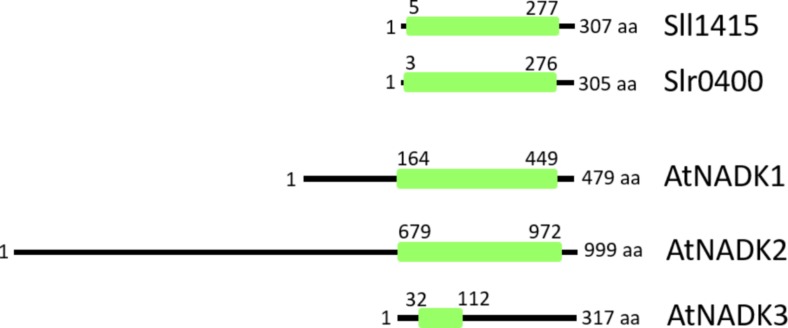
Comparison of primary structures of NADKs in *Synechocystis* sp. PCC 6803 (Sll1415 and Slr0400) and *Arabidopsis* (AtNADK1, AtNADK2, and AtNADK3). Conserved NADK regions (green box) span the entire length of the NADKs in *Synechocystis* sp. PCC 6803. In *Arabidopsis*, AtNADK1 and AtNADK2 possess N-terminal extensions, while, AtNADK3 possesses a C-terminal extension, compared to the various homologs. AtNADK1, At3G21070; AtNADK2, At1G21640; AtNADK3, At1G78590.

NAD kinase (NADK) phosphorylates NAD^+^ to NADP^+^,and is an activity that is present in all living organisms. In *Arabidopsis thaliana*, AtNADK2 produces NADP^+^ from NAD^+^ in the chloroplast. The *nadk2* knockout mutant exhibits a pale-green phenotype and is hypersensitive to environmental stresses (Chai et al., 2005; Takahashi et al., 2009). The primary sequence of *AtNADK2* includes a long N-terminal extension (compared to cyanobacterial homologs) that includes a chloroplast-targeting sequence as well as a calmodulin-binding site (Turner et al., 2004; Figure 2), and its additional N-terminal sequence exists in most plant chloroplast-localized species (Li et al., 2014). It is assumed that the NADP^+^ dynamics of the chloroplast are regulated by the enzyme activity of NADK2. On the other hand, AtNADK3, which localizes to the peroxisomal matrix (Waller et al., 2010), exhibits a different primary structure (Figure 2). Notably, NADK3 harbors a C-terminal extension (compared to homologs). To our knowledge, there have been no reports (to date) regarding the role of the NADK3 C-terminal region.

Based on BLAST searches of the cyanobacterial genomes available in the NCBI GenBank database, it was reported that almost all cyanobacteria possess two types of NADKs, despite the apparent lack of subcellular compartments within these cells (Gao and Xu, 2012). Consistent with these BLAST analyses, *Synechocystis* sp. PCC 6803 also harbors two NADK-encoding genes (*sll1415* and *slr0400*). Both proteins include conserved amino acid sequence motifs for NAD binding (GGDG) (Raffaelli et al., 2004; Mori et al., 2005) and ATP binding (NE/D) (Liu et al., 2005). Moreover, the Sll1415 and Slr0400 proteins do not harbor N- and C-terminal extensions like those found in *Arabidopsis* (Figure 2). Therefore, in cyanobacteria, two short-type NADKs appear to suffice for controlling the NADP^+^/NAD^+^ ratio in cells.

**FIGURE 3:**
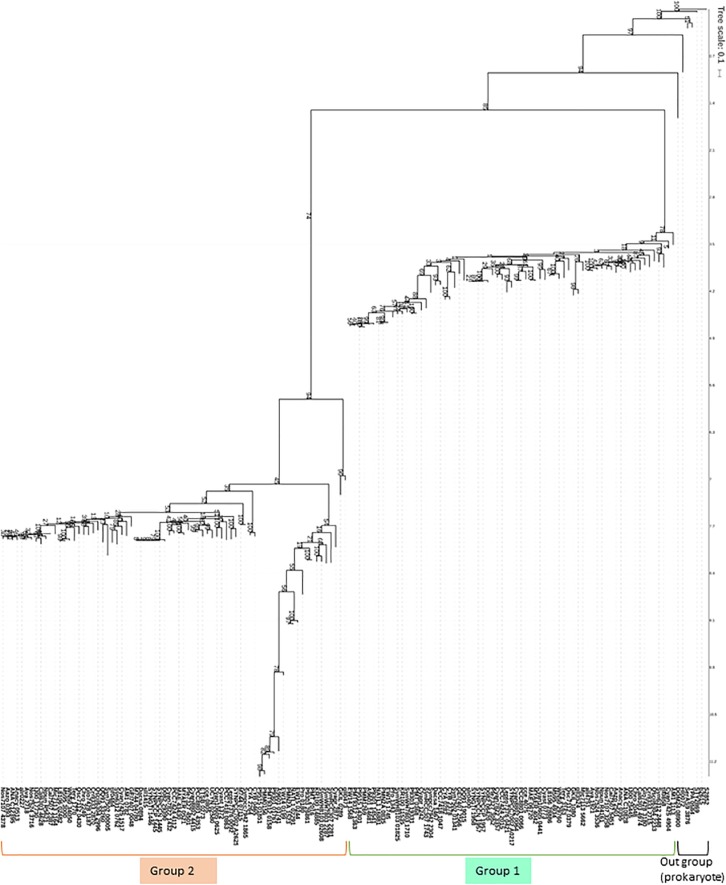
Phylogenetic tree of cyanobacterial NADKs. Most cyanobacteria possess two NADKs, representing one each of NADKs that group with Sll1415 (Group 2) or Slr0400 (Group 1). Bootstrap resampling analyses (100 times) with maximum likelihood was performed with PhyML (Guindon et al., 2010).

When the proteomes of the 72 species registered in cyanobase^1^ were searched for NADK homologs, 69 species had two typical NADKs, such that one of could be classified as being of the Slr0400 type (Group 1) or the Sll1415 type (Group 2). On the other hand, 2 species (*Atelocyanobacterium thalassa* and *Epithemia turgida*) harbored only one NADK, which belonged to the Sll1415 type (Group 2 in Figure 3 and Table 1). Interestingly, in the case of *E. turgida* spheroid body (*Et*SB), *Et*SB has lost photosynthetic ability and is metabolically dependent on its host cell (Nakayama et al., 2014). Additionally, *Atelocyanobacterium thalassa* was found to lack the oxygen-evolving photosystem II, RuBisCo (ribulose-1,5-bisphophate carboxylase-oxygenase that fixes CO_2_) and the tricarboxylic acid cycle (Zehr et al., 2008; Tripp et al., 2010). Additionally, *Atelocyanobacterium thalassa* has a symbiotic association with a unicellular prymnesiophyte (Thompson et al., 2012), prymnesiophyte receives fixed N in exchange for transferring fixed carbon to *Atelocyanobacterium thalassa*. Thus, we speculate that *slr0400* seems to be one of the key metabolic factors to understand the relationship between lost-photosynthesis, symbiotic and metabolically regulation in cyanobacteria. However, to our knowledge, there have been no reports (to date) regarding the role of the *slr0400*. Therefore, it remains why *Atelocyanobacterium thalassa* does not have NADK that group with Slr0400 in Figure 3. Based on our previous study, we speculate that *slr0400* may have key role in photosynthesis and suppression of the heterotrophic metabolism in *Synechocystis* sp. PCC 6803. Interestingly, *Synechococcus* sp. UTEX 2973 encodes two NADKs, both of which can be classified into Group 2 based on protein phylogeny (Figure 3). *Synechococcus* sp. UTEX 2973 is a fast-growing cyanobacterium that has been proposed as a candidate biomass-producing platform (Yu et al., 2015). Moreover, the markers associated with fast-growth in this species were determined (Ungerer et al., 2018); that analysis revealed that a NADK-encoding locus is one of the genetic factors that contributes to fast-growth.

**TABLE 1:** Cyanobacteria and putative NADKs used for the phylogenetic tree construction.

		
*Synechocystis* sp. PCC 6803	sII1415	slr0400
*Anabaena* sp. 90	ANA_C12395	ANA_C10828

Candidatus *Atelocyanobacterium thalassa* isolate ALOHA	UCYN_02580	

*Cyanothece* sp. PCC 8801	PCC8801_4353	PCC8801_0066
		
*Gloeobacter violaceus* PCC 7421	gll0473	gll3525
		
*Halothece* sp. PCC 7418	PCC7418_0863	PCC7418_1047
		
*Leptolyngbya* sp. PCC 7376	Lepto7376_0092	Lept7376_0421
		
*Microcoleus* sp. PCC 7113	Mic7113_1164	Mic7113_5662
		
*Nostoc* sp. PCC 7120	alr0227	all4751
		
*Prochlorococcus marinus* str. MIT 9301	P9301_01761	P9301_14541
		
*P. marinus* str. NATL2A	PMN2A_1523	PMN2A_0835
		
*Synechococcus* sp. UTEX 2973	M744_07115	M744_04780
		
*Synechococcus* sp. WH7803	SynWH7803_2287	SynWH7803_1710
		
*Synechocystis* sp. PCC 6803 substr. PCC-P	SYNPCCP_1445	SYNPCCP_1957
		
*Thermosynechococcus elongatus* BP-1	tlr0484	tll0858
		
Cyanobacterium endosymbiont of *Epithemia turgida* isolate EtSB Lake Yunoko	ETSB_0273	

*Orange and green columns correspond to Groups 1 and 2 (respectively) in Figure 3.*

These genetic results raise several questions, including the following: (1) How do the typical types of NADKs (Group 1 and Group 2 in Figure 3) contribute to NAD(P)(H) homeostasis in cyanobacteria? (2) How is the NAD(P)(H) balance regulated in cyanobacteria possessing unusual NADKs (notably including *Atelocyanobacterium thalassa*, *E. turgida* and *Synechococcus* sp. UTEX 2973)?

## Physiological Significance of NAD Kinases in *Synechocystis* sp. PCC 6803

Based on the phylogenetic tree (Figure 3), we hypothesized that the two types of cyanobacterial NADKs (Group 1 and Group 2) possess distinct roles in NAD(P)(H) metabolism. Therefore, we conducted metabolic analyses of *Synechocystis* sp. PCC 6803 harboring single mutations in either of the two NADK-encoding genes (Δ1415 or Δ0400). Notably, the wild-type (WT) strain and the single-mutant strains exhibited similar growth properties when cultured under photoautotrophic conditions (Ishikawa et al., 2016). However, Δ0400 (but not Δ1415) possessed a metabolic profile that differed from that of WT (Ishikawa et al., 2016). Specifically, Δ0400 showed metabolic changes in the levels of sugar phosphates (glucose-6-phosphate, ribulose-5-phosphate, 6-phosphogluconate, and fructose-6-phosphate), implying that the upper parts of the glycolytic pathway, the oxidative pentose phosphate pathway, and the Calvin cycle were affected by the disruption of *slr0400* (Ishikawa et al., 2016).

The unicellular cyanobacterium *Synechocystis* sp. PCC 6803 is a model organism that can grow under a wide range of conditions, including photoautotrophy, photoheterotrophy in the presence of glucose (that is, the ability to grow under illumination even in the absence of photosynthesis), and photomixotrophy in the presence of glucose. Gao and Xu (2012) showed that a *sll1415*-deficient mutant exhibits growth deficiency under photoheterotrophic conditions. Those authors reported that a *sll1415* null mutation affected the strain’s NADK activity and NADP(H) levels more severely than did a *slr0400* null mutation. Furthermore, we reported that the *sll1415*-deficient mutant showed a growth-impaired phenotype under growth conditions that would typically yield photomixotrophy (with 12-h light/12-h dark cycling), and under light-activated heterotrophic condition (LAHG) conditions (in which cells are maintained in darkness but exposed to light for 15 min every 24 h) (Ishikawa et al., 2019). Moreover, we showed that WT cells exhibited approximately 2.2- and 1.8-fold increases in the levels of NADP^+^ and NADPH (respectively; compared to 0 h) at 96 h after transfer from autotrophic to photoheterotrophic conditions. Furthermore, analysis of *sll1415* and *slr0400* mRNA levels in WT during the acclimation to photoheterotrophic conditions suggested dynamic changes in NADP^+^ biosynthesis. Based on detailed molecular analysis, we showed that Sll1415 appears to have a specific role in the oxidative pentose phosphate pathway, sustaining cell growth under photoheterotrophic conditions (Ishikawa et al., 2019).

The NADK-encoding gene *slr0400* also is an important factor in NAD(P)(H) homeostasis in cyanobacteria. When the *slr0400* mutant was grown under photoautotrophic conditions, the amount of glycogen was decreased compared to that in WT. Additionally, the levels of the *agp* transcript (encoding ADP-glucose pyrophosphorylase) decreased in Δ0400 grown under photoautotrophic conditions (Ishikawa et al., 2019). Furthermore, the metabolomic profile of Δ0400 was different from those of WT and Δ1415 when grown under photoheterotrophic conditions (Ishikawa et al., 2016; Ishikawa et al., 2019). Notably, the intracellular levels of major metabolites (such as fructose-6-phosphate and 6-phosphogluconate) were decreased in Δ0400 (Ishikawa et al., 2016). Interestingly, Δ0400 exhibited an increased NAD^+^/NADH ratio and a decreased NADPH level (compared to WT and Δ1415) under photoautotrophic conditions (Ishikawa et al., 2019). Based on these data, we hypothesize that Δ0400 cells are forced to remain in the heterotrophic mode even in the absence of glucose.

## Conclusion

NAD(P)(H) play an essential role in the metabolism of all organisms. However, it remains unclear how organisms maintain NAD(P)(H) homeostasis, and the physiological roles of NAD(P)(H) remain poorly characterized. Most cyanobacteria possess two structurally distinct NADKs, so it is assumed that these two protein types represent paralogs, that is, the two have distinct roles in cyanobacterial metabolism. As reviewed in this paper, one of the cyanobacterial NADKs (corresponding to Sll1415 of *Synechocystis* sp. PCC 6803) is critical for photoheterotrophic growth. However, it is not apparent why cyanobacteria, which have simple (prokaryotic) cell structures, have multiple NADKs, and the role of the separate NADK paralogs remain unclear. We believe that functional differences in the two typical NADKs must be crucial for cyanobacterial metabolic adaptation through control of NAD(P)(H) homeostasis. To elucidate the significance of NADKs in cyanobacteria, further characterization of the biochemical properties of NADKs, subcellular localizations of NADKs, and membrane permeability of NAD(P)(H) will need to perform.

## Author Contributions

YI wrote this review. Both authors approved the manuscript for publication.

## Conflict of Interest Statement

The authors declare that the research was conducted in the absence of any commercial or financial relationships that could be construed as a potential conflict of interest.

## Footnotes

1 http://genome.microbedb.jp/CyanoBase
